# Self-care practice and associated factors among Diabetes Mellitus patients on follow up in Benishangul Gumuz Regional State Public Hospitals, Western Ethiopia: a cross-sectional study

**DOI:** 10.1186/s13104-018-3939-8

**Published:** 2018-11-26

**Authors:** Segni Wanna Chali, Mohammed Hassen Salih, Addisu Taye Abate

**Affiliations:** Department of Medical Nursing, School of Nursing, College of Medicine and Health Sciences, University of Gondar, Gondar, Ethiopia

**Keywords:** Diabetes mellitus, Self-care practice, Benishangul Gumuz

## Abstract

**Objective:**

The aim of this study was to assess the self-care practice among patients with diabetes and their associated factors in Benishangul Gumuz Public Hospitals, Western Ethiopia, 2018.

**Result:**

Out of the total 399 selected patients, 383 were participated in the study with a response rate of 96%. From 383 respondents, 45.7% had poor diabetes self-care practice. Unable to read and write (AOR = 3.63, 95% CI 1.33–9.89, p = 0.011), never had a diabetic health education (AOR = 4.09, 95% CI 1.89, 8.84, p = 0.000), not having glucometer (AOR = 2.66, 95% CI 1.30, 5.46 p = 0.007), poor diabetic knowledge (AOR = 5.01, 95% CI 2.44, 10.28, p = 0.000), poor self-efficacy (AOR = 3.00, 95% CI 1.76, 5.11, p = 0.000) and not having social support (AOR = 1.84, 95% CI 1.08, 3.13, p = 0.023) were significantly associated with poor self-care practice of diabetes patients. These findings request for the need of integrated interventional management approach, which will improve the health and quality of life of the diabetes patients.

## Introduction

Diabetes mellitus (DM) is a metabolic disorder of multiple etiologies characterized by increased level of glucose in the blood with disturbances of carbohydrate, fat and protein metabolism resulting from defects in insulin secretion, insulin action, or both [1]. It is a complex, chronic illness demanding continuous medical and self-care [2].

Diabetes is a global health problem targeted for action and currently increasing both in the number of cases and the prevalence [3, 4]. According to the International Diabetes Federation (IDF) 2017 reports, more than 425 million people worldwide are reported as diabetic patients and nearly 80% of them are living in low and middle-income countries including Ethiopia. Globally, more than 212 million people with diabetes are not aware of their disease and there are above 352 million people with impaired glucose tolerance (IGT) [5], which puts them at high risk of developing diabetes and its complications like cardiovascular diseases, stroke, kidney failure, foot ulcer, visual impairment and nerve damage [3, 5, 6].

Diabetes self-care is important to keep the disease under control, It includes performing activities such as healthful eating, regular physical activity, foot care, medication adherence, and self-monitoring of blood glucose (SMBG) [6]. However, it is highly challenging since many people with diabetes may have contact with a healthcare professional for a total of a few hours per year and factors such as diabetes knowledge, physical activities, social support and self-efficacy can affect the self-care practice [7, 8].

In Ethiopia, diabetes becomes a fast-growing and more common chronic illness, in which > 2.135 million people are expected to be diabetic patients and it becomes the most common cause of admission which fasten the development of complications like heart attack and strokes; as a result it shortens an individual lifespan by 10–15 years. Despite this, the feature of self-care practices towards diabetes was not adequate [9–11]. Diabetes has a great burden on the quality of life and socioeconomic structures of the affected individuals, their families, and the country’s economic status. Countries like Ethiopia, where the resources are limited, and treatment costs of the disease are constantly increasing, good adherence to diabetic self-care practice may result in better economic and therapeutic outcomes [6, 12, 13]. Although such studies are important in such resource-limited areas, to realizing the various complex nature of the problem and to individualize, integrate the clinical approach that will enhance the diabetic self-care practice utilization [6, 7, 14], there is no study conducted in Benishangul Gumuz public hospitals regarding self-care practice on diabetic patients; few studies conducted in developing countries have discrepancy on self-care practice among diabetes patients and all of the available literatures in Ethiopia were limited in addressing factors that influence self-care practice. Therefore, this study aimed to assess self-care practice and associated factors among diabetic patients in Benishangul Gumuz public hospitals, Western Ethiopia.

## Main text

### Study design and setting

An Institution based cross-sectional study on 383 patients was conducted from March 15-April 15/2018 G.C. at Benishangul Gumuz public hospitals (Assosa and Pawi Hospitals), Western Ethiopia.

### Sample size determination and procedure

The source population were all diabetic patients who were on diabetic follow up at Benishangul Gumuz public Hospitals. All diabetic patients aged ≥ 18 years and who have been on regular follow for DM were included and patients who were critically ill, with severe mental illness or who were unable to provide the required information by themselves were excluded. Single population proportion formula was used with the assumption of 95% confidence interval, 5% margin of error, (55%) proportion of good diabetic self-care practice [10], and 5% for possible non-response was taken to determine a final sample size of 399. Systematic random sampling technique was utilized; sampling interval “K” value was calculated as K = N/nf, where N = the expected Number of diabetes patients per month = 798 and nf = final sample size = 399 which gives a sampling interval of two. Thus, using patients’ record order which was listed in follow up appointment as a sampling frame, study subjects were selected in every 2 number intervals until to reach the total sample size and the first participant was selected by lottery method.

### Data collection method and survey instrument

Data collection was performed by four BSc nurses through an interviewer-administered questionnaire. The data collectors were properly trained on the instrument and ways of approaching the patients and how to obtain permission for an interview prior to the data collection process for 3 days. Initially, the questionnaire was translated from English to the official/local language of the region (Amharic); then it was re-translated to English language to ensure consistency. The data collection tool was pretested on 19 adult diabetic patients who were not included in the final analysis and relevant modifications were done before the actual data collection period. The tool had six sections: Section one: contain socio-demographic variables. Section two: includes clinical characteristics. Section three: contain the summary of diabetes self-care activities (SDSCA) questionnaire, which was adopted from a validated SDSCA measure revised from seven studies result [15]. The SDSCA tool is frequently used to measure the domains of diabetic self-care practices: general diet, specific diet, exercise, medication, SMBG and foot care. The overall mean score was calculated by summation of the mean score of each domain divided by the sum of the number of questions under each scale. After calculating the overall mean score, it was classified as having good self-care practice if respondents score ≥ 3 or poor if scored < 3. Section four: Contain the diabetes knowledge test adopted from previously validated tools of the Revised Brief Diabetes Knowledge Test (DKT2) [16]. Section five: Contain the self-efficacy for diabetes self-care tests, which was adopted from previously validated tools of the Diabetes Empowerment Scale-Short Form (DES-SF) questioners [17]. Section six: Contain the social support questions, adopted from previously validated tools of Brief Scale for Social Support questioners. It contains 9 items rated on a 5-point Likert-type scale from none (1), almost none (2), some (3), a lot (4), and very much (5) [18].

### Statistical analysis

All the data was checked visually, coded and entered into Epi Info version 7 and exported to Statistical Package for Social Sciences (SPSS) version 21 for analysis. Frequencies, percentages, summary statistics like mean and standard deviation were examined to describe the data. Binary logistic regression was run to see the crude significant relations of each independent variable with the poor diabetes self-care practice. Then by selecting variables with p-value ≤ 0.2 in bi-variable logistic regression analysis were again entered into multivariable logistic regressions. Finally, significant factors were identified based on adjusted odd ratio (AOR) included in 95% confidence level at p-value ≤ 0.05. Then, the data was described and presented using narrative text, chart and tables.

## Results

### The Socio-demographic characteristics

Out of the total 399 study participants planned, 383 were participated in the study with a response rate of 96%. More than half (54.6%) of them were male. About 95(24.8%) of the study participants were in the age group of 40 to 49 years old and the mean age of the participants was 44.5 (± 14.9) years old (Table 1).

**Table 1:** Socio demographic characters of diabetes patients on follow up at selected hospital in Benishangul Gumuz regional state, Western Ethiopia, 2018. (n = 383)

S.no	Variables	Frequency	Percentage	Remarks
1	*Sex*			
	Male	209	54.6	
	Female	174	45.4	
2	*Age*			Mean = 44.54 (SD = 14.882)
	≤ 29	60	15.7	
	30–39	90	23.5	
	40–49	95	24.8	
	50–59	67	17.5	
	≥ 60	71	18.5	
3	*Level of education*			
	Can’t read and write	86	22.5	
	Read and write	85	22.1	
	Primary school	89	19.6	
	Secondary school	95	24.8	
	Above 12	42	11.0	
4	*Religion*			
	Orthodox	135	35.2	
	Muslim	126	32.9	
	Protestant	72	18.8	
	Others	50	13.1	
5	*Ethnicity*			
	Amhara	103	26.9	
	Oromo	79	20.6	
	Shinasha	59	15.4	
	Berta	49	12.8	
	Gumuz	30	7.8	
	Others	63	16.4	
6	*Marital status*			
	Single	85	22.2	
	Married	226	59.0	
	Divorced	41	10.7	
	Widowed	31	8.1	
7	*Occupation*			
	Student	58	15.1	
	Employed	121	31.6	
	Unemployed	71	18.5	
	House wife	47	12.3	
	Farmer	45	11.8	
	Merchant	41	10.7	
9	*Residency*			
	Urban	272	71.0	
	Rural	111	29.0	

### Health-care related factors

More than half, 222(58.0%) participants had type two diabetes, and the mean diabetic duration of the participants were 4.55 (± 3.381) years. Of the respondents, nearly three-fourth (79.9%) of them had no glucometer, only 54(14.1%) had a family history of diabetes and majority of them (82.8%) had no additional chronic illnesses. In addition, about (17.0%) respondents never had a diabetic health education (Table 2). Generally, More than half, 208 (54.3%) of respondents had good diabetic self-care practice.

**Table 2:** Health-care related factors of diabetes patients on follow up at selected hospital in Benishangul Gumuz region, Western Ethiopia, 2018(n = 383)

No	Clinical and related characteristics	Frequency	Percentage (%)	Remark
1	*Type of DM*			
	Type 1	73	19.1	
	Type 2	222	58.0	
	I don’t know	88	22.9	
2	*Duration of DM *			
	≤4 years	227	59.3	
	5–9 years	109	28.5	
	10–14 years	32	8.4	
	≥15 years	15	3.9	
3	*Family history of DM*			
	Yes	54	14.1	
	No	188	49.1	
	I don’t know	141	36.8	
4	*Additional chronic illness*			
	Yes	66	17.2	
	No	317	82.8	
5	*Current treatment*			
	Insulin injection	101	26.4	
	Oral anti hyperglycemic	226	59.0	
	Both	56	14.6	
6	*Diabetic health education*			
	Never	65	17.0	
	Sometimes	170	44.4	
	Regularly	148	38.6	
7	*Having glucometer*			
	Yes	77	20.1	
	No	306	79.9	
8	*Knowledge*			
	Poor knowledge	113	29.5	
	Moderate knowledge	159	41.5	
	Good knowledge	111	29.0	
9	*Social support*			
	Good social support	218	56.9	
	Poor social support	165	43.1	
10	*Self-efficacy*			
	Good self-efficacy	203	53.0	
	Poor self-efficacy	180	47.0	

### Factors associated with diabetes self-care practice

In the present study, the odds of respondents who were unable to read and write was 3.6 times more likely (AOR = 3.63, 95% CI 1.33–9.89, p = 0.011) than that of secondary and above educational level. For respondents who never had a diabetes health education, the odds of having poor self-care practice was 4 times (AOR = 4.09, 95% CI 1.89, 8.84, p = 0.000) than those who had regular diabetes health education. For not having a glucometer, the odds of having poor self-care practice was 2.6 times (AOR = 2.66, 95% CI 1.30, 5.46, p = 0.007) that of who had a glucometer. For Respondents who had poor diabetes knowledge, the odds of poor diabetes self-care practice was 5 times (AOR = 5.01, 95% CI 2.44, 10.28, p = 0.000) that of who had good diabetes knowledge. For Respondents who had poor diabetes self-efficacy, the odds of poor self-care practice was 3 times (AOR = 3.00, 95% CI 1.76, 5.11, p = 0.000) that of who had good self-efficacy. The odds of respondents who had no social support’s poor self-care practice was 1.8 times (AOR = 1.84, 95% CI 1.08, 3.13, p = 0.023) that of who had social support (Table 3).

**Table 3:** Factors associated with self-care practice of diabetes patients on follow up in Benishangul Gumuz Regional State Hospitals, Western Ethiopia, 2018, (n = 383)

Variables	Self-care practice		COR (95%CI)	AOR (95%CI)	p-value
	Good	Poor			
Sex					
Male	125	84	1.00	1.00	
Female	83	91	1.63 (1.09, 2.45)	2.18 (1.26, 3.75)	0.005*
Age					
18–29	30	30	1.00	1.00	
30–39	49	41	0.84 (0.44, 1.61)	0.56 (0.19, 1.61)	0.28
40–39	61	34	0.56 (0.29, 1.08)	0.43 (0.14, 1.34)	0.15
50–59	37	30	0.81 (0.40, 1.63)	0.59 (0.17, 2.01)	0.40
≥ 60	31	40	1.29 (0.65,2.57)	0.95 (0.27, 3.33)	0.93
Level of education					
Can’t read and write	34	52	4.89 (2.13,11.24)	3.64 (1.34, 9.89)	0.01*
Read and write	38	47	3.96 (1.73, 9.07)	3.22 (1.17, 8.82)	0.02*
Primary school	41	34	2.65 (1.14, 6.17)	1.88 (0.68, 5.23)	0.23
Secondary school	63	32	1.63 (0.71, 3.72)	1.65 (0.61, 4.47)	0.32
Above 12(tertiary)	32	10	1.00	1.00	
Marital status					
Single	44	41	0.44 (0.19, 1.05)	0.79 (0.22,2.79)	0.72
Married	134	92	0.33 (0.15, 0.73)	0.88 (0.30,2.58)	0.82
Divorced	20	21	0.50 (0.19, 1.32)	0.82 (0.23,2.89)	0.76
Widowed	10	21	1.00	1.00	
Religion					
Orthodox	72	63	1.01 (0.62, 1.64)	1.03 (0.54, 1.94)	0.94
Muslim	67	59	0.61 (0.34,1.10)	0.99 (0.36, 1.60)	0.48
Protestant	47	25	1.46 (0.76, 2.79)	1.08 (0.43, 2.32)	1.00
Others	22	28	1.00	1.00	
Ethnicity					
Amhara	54	49	1.134 (0.609, 2.129)		
Oromo	49	30	0.769 (0.390, 1.501)		
Shinasha	30	29	1.208 (0.593, 2.464)		
Berta	28	21	0.938 (0.441, 1.991)		
Gumuz	12	18	1.875 (0.775, 4.536)		
Others	35	28	1.00		
Occupation					
Student	33	25	1.00		
Employed	66	55	1.18 (0.52, 2.67)		
Unemployed	36	35	1.30 (0.63, 2.68)		
House wife	25	22	1.52 (0.70, 3.32)		
Merchant	25	16	1.38 (0.59, 3.22)		
Farmer	23	22	1.50 (0.63, 3.52)		
Residency					
Urban	163	109	1.00	1.00	
Rural	45	66	2.19 (1.40, 3.50)	1.65 (0.91, 2.99)	0.10
Type of DM					
Type 1	40	33	1.00	1.00	
Type 2	138	84	0.74 (0.43, 1.26)	1.12 (0.44, 2.88)	0.81
I don’t know	30	58	2.34 (1.24, 4.43)	2.49 (0.87, 7.12)	0.09
Duration of DM (years)					
0–4	119	108	0.79 (0.28, 2.26)		
5–9	62	47	0.66 (0.22,1.96)		
10–14	20	12	0.53 (0.15, 1.82)		
≥ 15	7	8	1.00		
DM family history					
Yes	36	18	1.00	1.00	
No	101	87	1.72 (0.91, 3.25)	1.37 (0.61, 3.09)	0.45
I don’t know	71	70	1.97 (1.02, 3.80)	1.48 (0.63, 3.45)	0.37
Comorbidity					
Yes	41	25	0.68 (0.39, 1.17)	0.57 (0.28, 1.16)	0.12
No	167	150	1.00	1.00	
Current treatment					
Insulin injection	53	48	0.91 (0.47,1.75)		
Oral DM medications	127	99	0.78 (0.43, 1.40)		
Both	28	28	1.00		
DM health education					
Never	20	45	4.84 (2.58, 9.08)	4.10 (1.90, 8.84)	0.000*
Sometimes	87	83	2.05 (1.30, 3.24)	1.08 (0.79, 2.49)	0.25
Regularly	101	47	1.00	1.00	
Having glucometer					
Yes	58	19	1.00	1.00	
No	150	156	3.18 (1.81, 5.58)	2.66 (1.30, 5.47)	0.007*
Diabetic knowledge					
Poor	36	77	5.52 (3.11, 9.79)	5.02 (2.45, 10.28)	0.000*
Moderate	92	67	1.88 (1.12, 3.16)	1.86 (0.98, 3.55)	0.060
Good	80	31	1.00	1.00	
Social support					
Good	140	78			
Poor	68	97	2.56 (1.69, 3.88)	1.85 (1.09, 3.13)	0.023*
Self-efficacy					
Good	137	66	1.00		
Poor	71	109	3.19 (2.10, 4.85)	3.01 (1.76, 5.12)	0.000*

*COR* crude odd ratio, *AOR* adjusted odd ratio, *DM* diabetes mellitus

NB: variables having a (p ≤ 0.2) in bi variable (unadjusted) analysis included in the multivariable (adjusted) analysis. * Satistically significant at p-value ≤ 0.05

## Discussion

In this study, the magnitude of overall poor self-care practice was 45.7% (95% Cl 40.9–51%); which is consistent with studies conducted in Dilla University Hospital [19], Nekemte referral Hospital [10], Mekele and Ayder Referral Hospital, Ethiopia [20], and India [21] which were (44%, 45%, 49% and 50.5%) respectively. However, it is higher than a study conducted in Addis Ababa (39.7%) [21]. In the contrary, the finding is lower than the study conducted in Kenya, Felege Hiwot and Harar Hospital, Ethiopia (59%, 63.2% and 60.8%) respectively [9, 22, 23]. The possible reasons for this difference could be the difference in the sources of information, socio-cultural variation, inadequate access of the glucometer, inadequate health education towards self-care practice and educational level of the study participants.

In the present study, those who were unable to read and write were 3.6 times more likely to have poor self-care practice than those who were Grade 12 and above. Similar findings were observed in a study conducted in Harrari [9], Jimma [25] and FelegeHiwot hospital, Northwest Ethiopia [24]. Moreover, diabetes health education had a preventive effect against poor self-care practice and this finding was supported by findings from Addis Ababa and Bahir Dar hospitals [21, 23]. The possible reason behind this finding might be the diabetes education given by health professionals increased the interest of patients on their own health and created awareness that enhances the self-care practice.

Not having glucometer was also significantly associated with poor self-care practice; this finding is comparable with a study conducted in Anand District of Gujarat [13]. The possible reason could be having a glucometer at home may reinforce to monitor their blood glucose level regularly. Respondents who had poor diabetes knowledge were also 5 times more likely to have poor self-care practice than those who had good diabetes knowledge. This finding was supported with the study conducted in Nekemte, Indian and Bangladesh [9, 10, 19].

Additionally, respondents who had no diabetes self-efficacy were more likely to have poor diabetes self-care and which was similar with studies conducted in Malaysia and Omani [25, 26]. This study finding also showed that social support was one factor that affects self-care practice in diabetes patient; in which participants who had no social support were more likely to have poor self-care practice than those who had social support. This finding was supported by the study done in Jimma [25], Anand District of Gujarat and India [13, 21]. The possible reason for this might be due to having social support may be considered as a guiding force that reinforce individuals for the better self-care practice.

## Conclusion

Generally, the finding of this study revealed that a significant number of diabetes patients had a low level of self-care practice. These findings request for the need of integrated interventional management on diabetes, which will increases health and wellbeing of the patients. Therefore, in order to improve diabetes self-care practice; different stakeholders including Hospitals, health professionals, health programmers, and different non-governmental organizations should give emphasis on linking diabetic patients to different supporting social groups, improving knowledge through health education and providing self-monitoring glucometer for those individuals who are unable to buy by themselves.

## Limitation

Since the study was a cross-sectional study, which was poor in establishing a temporal relationship and the data collection method was self-report rather than direct observation of patient’s self-care practices.

## Abbreviations

ADA: American diabetic association
AOR: adjusted odds ratio
BGRS: Benishangul gumuz regional state
BSC: Bachelor of Science
CL: confidence level
COR: crude odds ratio
CSA: central statistical agency
DC: data collectors
DES-SF: diabetic empowerment scale short form
DKT2: the revised brief diabetic knowledge test
DM: diabetes mellitus
IDF: International diabetic federation
IGT: impaired glucose tolerance
Epi info: statistical package for epidemiological information analysis
ETB: Ethiopian Birr
FMOH: Federal ministry of health
MSC: Masters of Science
OPD: outpatient department
OR: odds ratio
PI: Principal Investigator
SDSCA: summary of diabetic self-care activities
SMBG: self-monitoring blood glucose
SPSS: statistical package for social science
T2: type two
